# On the Prevention of Mammary Abscesses by the Application of the Principle of Rest

**Published:** 1875-08

**Authors:** 


### ON THE PREVENTION OF MAMMARY ABSCESSES BY THE APPLICATION OF THE PRINCIPLE OF REST.

Dr. W. Bathurst Woodman read a paper on this subject before the Obstetrical Society of London (*Med. Times and Gaz.*, Jan. 16, 1875). He had been struck with the rarity of mammary abscesses in animals, notwithstanding the forced abstinence from suckling which cats and dogs undergo for the drowning of their progeny, and in spite of the great distension of the udders of cows, mares, and other animals when driven to market, or for other reasons separated from their young. Acting upon this suggestion, he carefully abstained from those manipulations and questionable "gentle" frictions which have so long been customary in such cases, and with the most satisfactory results. Where an abscess was threatening, in place of employing liniments he enjoined perfect rest, the avoidance of all frictions and rough handling, and of suckling for a time—if possible from both breasts, but at all events from the most implicated; the horizontal position, careful application of strips of isinglass, soap, or lead plaster, or of an air-cushion with a hole in the centre, or of bandages taking their purchase from the opposite shoulder. In addition to these measures he employed preparations of opium, belladonna, or chloroform, applied in compresses, or ice, moist warmth, and leeches; the local congestion being also relieved by diaphoretics, diuretics, and aperients—belladonna, iodide of potassium, and sedatives being given if requisite. Illustrative cases of this method of treatment were given, exemplifying its advantages.

Dr. Barnes observed that the principle of rest had long been applied to the treatment of inflammation of the breast. He himself had learned the value of it from Trousseau, when a student in Paris thirty years ago. That admirable physician taught and illustrated it with great earnestness. He placed the breast at perfect rest by carrying straps of leather spread with *emplater de vigo* all around it, so as to lift it well up and exert constant support on the vessels. Thus œdema was prevented, and engorgement soon subsided. It must, however, be remembered that this form of pressure was ill borne in the first inflammatory stage. It was chiefly serviceable when suppuration had taken place and the abscess had been opened; the sac then rapidly closed. In the earlier stage he had seen leeches do excellent service. The pressure must be lighter.

Dr. Ashburton Thompson said there were two modes of treatment not referred to in this paper—the administration of tincture of aconite, and total abstention from fluids during the necessary number of days. By giving minim doses of aconite every hour he had succeeded in cutting short inflammation of the breast which there was no doubt would otherwise have run on to suppuration very frequently; indeed, in three cases out of four. In cases of stillbirth he had hitherto found abstention from fluids sufficient in every case to avoid every kind of mammary disturbance. Ice was allowed in moderate quantity, and no other fluid, from the time of delivery until the fourth or fifth day, when the breasts generally return to their normal state of quiescence. He had had two cases recently in which this method of treatment had been perfectly successful. The deprivation of fluid caused but little distress.

Dr. Braxton Hicks thought the principle of rest had been gradually coming upon us for years, friction only being resorted to among the poor and ill-educated. Surgery at the present day was all tending to quietude. Manipulations only led to suppuration, and often produced the extra amount of stimulation required to set it up.

Dr. Murray observed that the application of belladonna plaster was of great service, keeping the arm at the same time fastened to the side. In some instances a slight process of friction upwards was productive of good.

Dr. Matthews, whilst heartily assenting to Dr. Woodm'n's views, thought that the public also had largely endorsed his practice, since he had observed that it was a very common proceeding to apply a large lead plaster (spread upon leather) to the breast in cases where it becomes necessary to get rid of the milk; this of course rendered friction and all meddling impossible. He had found two large and suitable handkerchiefs suitably applied—one by way of sling across the neck under the breast, the other in exactly the reverse way, over the breast, and tied around the body so as to include the breast between them, interposing a large pad of cotton-wool—to constitute a very efficient mode of applying pressure.

Dr. Edis remarked that the chief thing to be remembered was to limit the supplies, to act on the bowels, and to insure perfect rest to the mammæ. He was accustomed to order a belladonna plaster to be applied to the mammary region within twenty-four hours of delivery, thus exercising pressure as well as arresting the secretion of milk. Abstinence from fluids and great moderation in diet were enjoined for the first few days, an aperient mixture of sulphate of magnesia and iodide of potassium being given twice or thrice daily to relieve the bowels. The shoulders should be raised, and the arms kept perfectly quiet; the upper part of the chest being only lightly covered; any friction or drawing of the breasts being strictly prohibited. Where this method had been adopted he had never seen a single instance of mammary abscess. An evaporating lotion continuously applied to the mammæ was in some instances sufficient to prevent the secretion of milk ; but the pressure obtained from the plaster was of great service, and effectually prevented the employment of any friction.

---

*Damiana—A New Remedy.*—Dr. J. J. Caldwell, of Baltimore, in the May number of the *Virginia Medical Monthly*, describes the effects of a Mexican plant, with the above name, which would appear to possess more extraordiary aphrodisiac power than any other known agent. In a nnmber of cases of exhaustion and impotency in both sexes, he administered a strong tincture, in tablespoonful doses, three or four times a day, with results very well marked occurring in two to six weeks. One case was a man aged seventy, another was a female aged forty, and a third was a man who had greatly impaired his genito-unrinary organs by excessive drinking. In cases of healthy men, on whom he tried the article for experiment, the sexual desire became almost ungovernable. The remedy can be obtainad at any one of our apothecaries.—*Balt. Phys. & Surg.*

---

*Cure of Vesico-Vaginal Fistula by Caustics*—Many distinguished Italian and Belgian surgeons maintain that vesico-vaginal fistula may often be cured by cauterization. Dr. L. de Lorge has brought before the Medical Society at Ghent a case which he cured by this method, and which was watched by Prof. van Wetter. Three cauterizations, on the 20th of November and on the 4th and 18th of December, sufficed to completely close the fistula, which was at first one centimetre in diameter. The first application was made within a month of the delivery, at which the accident was produced. Dr. de Lorge employed caustic potass, and applied it very freely all round the edges of the fistula. After the application the walls of the vagina were separated by a piece of sponge. The patient did not suffer much pain, and within two days the dribblings of urine was diminished. So the cure progressed gradually but surely.—*The Doctor.*

